# Substantiate a read-across hypothesis by using transcriptome data—A case study on volatile diketones

**DOI:** 10.3389/ftox.2023.1155645

**Published:** 2023-05-03

**Authors:** Christina Drake, Matthias M. Wehr, Walter Zobl, Jeannette Koschmann, David De Lucca, Britta A. Kühne, Tanja Hansen, Jan Knebel, Detlef Ritter, Jan Boei, Harry Vrieling, Annette Bitsch, Sylvia E. Escher

**Affiliations:** ^1^ Fraunhofer Institute for Toxicology and Experimental Medicine, Chemical Safety and Toxicology, Hannover, Germany; ^2^ GeneXplain GmbH, Wolfenbüttel, Germany; ^3^ Medizinische Hochschule Hannover, Hannover, Germany; ^4^ Leiden University Medical Center, Leiden, Netherlands

**Keywords:** read across, new approach methodology, transcriptomics, TempO-seq, PBEC, protein network analysis

## Abstract

This case study explores the applicability of transcriptome data to characterize a common mechanism of action within groups of short-chain aliphatic α-, β-, and γ-diketones. Human reference *in vivo* data indicate that the α-diketone diacetyl induces bronchiolitis obliterans in workers involved in the preparation of microwave popcorn. The other three α-diketones induced inflammatory responses in preclinical *in vivo* animal studies, whereas beta and gamma diketones in addition caused neuronal effects. We investigated early transcriptional responses in primary human bronchiolar (PBEC) cell cultures after 24 h and 72 h of air-liquid exposure. Differentially expressed genes (DEGs) were assessed based on transcriptome data generated with the EUToxRisk gene panel of Temp-O-Seq^®^. For each individual substance, genes were identified displaying a consistent differential expression across dose and exposure duration. The log fold change values of the DEG profiles indicate that α- and β-diketones are more active compared to γ-diketones. α-diketones in particular showed a highly concordant expression pattern, which may serve as a first indication of the shared mode of action. In order to gain a better mechanistic understanding, the resultant DEGs were submitted to a pathway analysis using ConsensusPathDB. The four α-diketones showed very similar results with regard to the number of activated and shared pathways. Overall, the number of signaling pathways decreased from α-to β-to γ-diketones. Additionally, we reconstructed networks of genes that interact with one another and are associated with different adverse outcomes such as fibrosis, inflammation or apoptosis using the TRANSPATH-database. Transcription factor enrichment and upstream analyses with the geneXplain platform revealed highly interacting gene products (called master regulators, MRs) per case study compound. The mapping of the resultant MRs on the reconstructed networks, visualized similar gene regulation with regard to fibrosis, inflammation and apoptosis. This analysis showed that transcriptome data can strengthen the similarity assessment of compounds, which is of particular importance, e.g., in read-across approaches. It is one important step towards grouping of compounds based on biological profiles.

## 1 Introduction

In Europe, human risk assessment is undergoing a paradigm shift towards the integration of mechanistic data from primarily human *in vitro* and *in silico* models, referred to as new approach methodologies (NAMs). The use of mechanistic evidence, has the potential to significantly improve risk assessment and reduce uncertainties and, thus, seems promising in many respects. A better understanding of the biological mechanisms causing adverse toxicological effects will help to identify new ways of prevention and thus risk management. NAMs such as high-content or high-throughput data will also enable faster and more efficient assessment, providing data that is directly relevant to humans.

In line with animal welfare and the 3R principle, several European agencies, such as EFSA (EFSA, 2021), EMA (European Medicines Agency, 2021), have expressed their intention to reduce, refine and replace animal testing as far as possible within in the next decade by integration of NAMs. In other regulatory areas, e.g., for cosmetics, animal testing is banned (Pistollato et al., 2021) or, as under REACH, is only considered appropriate when all other options have been explored (European Commission. Regulation, 2019).

A challenge for the implementation of NAMs into regulatory decision-making lies in the development of concepts and overarching frameworks for the interpretation and integration of NAM data (Knight et al., 2021).

Case studies on the integration of mechanistic data, e.g., in the context of Adverse Outcome Pathways (AOPs) or Integrated Assessment and Evaluation (IATA), help to develop new assessment concepts, learn about their advantages and limitations, address uncertainties and thus advance their implementation in risk assessment.

In line with this idea, read-across is an established approach that is well suited to evaluate the new methods and gain more confidence in the use of NAMs as they offer the possibility to compare the traditional approach based on *in vivo* animal data with evidence from NAM data (Escher et al., 2019).

Read-across is a well-known approach in chemical risk assessment to estimate the toxicity of a target compound for which substance-specific experimental data are not available. It is of particular importance for high tier endpoints like long-term toxicity caused by low level exposure to substances occurring at workplace or in household products (Ball et al., 2016). The read-across assessment framework (ECHA, 2017) provides guidance on read-across approaches using traditional preclinical animal studies, while similar guidance for the integration of new approach methods such as “omic” data is missing to date.

The read-across approach assumes that substances with similar toxicokinetic and toxicodynamic properties will cause similar toxicological effects in an exposed organism or follow a consistent trend (ECHA, 2017). The most challenging step in the read-across assessment is usually to justify the similarity of the source to the target compound(s), as often only the structural and physicochemical properties of the target compound are known. Many read-across assessments are therefore often not accepted by authorities, as they fail to provide sufficient evidence on share toxicodynamic and kinetic properties of the grouped compounds (Ball et al., 2016).

The starting point of most read-across assessments is, therefore, an assessment of shared structural and physicochemical properties leading to the identification of an initial list source compounds. Subsequently, the available *in vivo* endpoint data are used to conclude on shared toxicodynamic properties within the grouped compounds. As *in vivo* studies are usually descriptive and do not provide insights into the mechanisms leading to the observed toxic effects, a shared toxicological effect pattern can be justified but usually not a shared mode of action. The assessment is complicated by the fact that often study results from different laboratories need to be compared. These studies differ with regard of study design (e.g., species tested, study size, dose selection and dose intervals), which leads to some variability in the observed apical findings (Ball et al., 2016; Judson et al., 2016).

A more precise assessment of shared toxicodynamic properties within the grouped compounds, based on, e.g., the description of their molecular mechanisms, will significantly reduce the uncertainty in read-across assessments. Few case studies have been published in which NAMs, like *in vitro* and *in silico* models, are used to substantiate the assessment of shared mode of actions in read-across approaches (Pawar et al., 2019; Grimm et al., 2019; Escher et al., 2019).

Although transcriptome data provide information on the perturbation of gene activities (Escher et al., 2019), it is still a challenge to infer the associated cellular processes and mechanisms that may finally result in the development of diseases or adverse outcomes (Sebastian-Leon et al., 2014). These data are, therefore, to date seldomly used in regulatory decision making (Karahalil, 2016). In addition, several analytical and technical challenges have been reported, including a large variety of alternative analysis strategies and the complexity of the data. Also, conceptual frameworks for the integration of omic data into regulatory hazard assessment are lacking, as there is no consensus to date on the approach to assess pathways and adverse effects, derive benchmark doses for gene perturbations and pathway analyses, and quantify uncertainties. Case studies with relevant regulatory problem formulations can serve as a tool to gain more confidence into new approaches such as omic supported hazard assessments and by this help to close these conceptual data gaps.

The read-across case study presented here investigates the use of transcriptome data for the evaluation of (dis)similar mechanism of action between grouped compounds. For this purpose, a group four α-diketones is analysed, of which three compounds are suspected to induce pulmonary fibrosis (bronchiolitis obliterans) in rodents (Brass and Palmer, 2017). In addition to these structurally very similar α-diketones, one structurally related β- and one γ-diketone are included into the assessment to identify potential (dis)similarities between these volatile diketone compounds. One compound with an entirely different mode of action, tunicamycin, is tested to be able to identify to some extent normal adaptive cellular responses. Two ketones, butanone, and acetone, are tested as negative compounds because both compounds did not induce any adverse effect in preclinical rodent studies with repeated exposure (Chen and Hee, 1995).

## 2 Materials and methods

### 2.1 Chemicals

Chemicals were purchased at the highest purity available. α-diketones: 2,3-Butandione (diacetyl, purity 99.5%, Lot No. SHBG3507V, Sigma B85307-100 ML), 2,3-Pentanedione (purity 97.1%, Lot No. MKBB7504V, Sigma 241962-25G), 2,3-Hexanedione (purity 96.0%, Lot No. MKBV4849V, Sigma W255807-250G-K), and 3,4-Hexanedione (purity 96.7%, Lot No. STBF5886V, Sigma 306932-25G), one β-diketone: acetylacetone (2,4-Pentanedione, purity 99.8%, Lot No. STBF8568V, Sigma 10916-1 KG), one γ-diketone: acetonylacetone (2.5-Hexanedione, purity 99.7%, Lot No. WXBC2583V, Sigma 165131-25G), butanone (purity 99.9%, Lot No. MKBV9520V, Sigma W217012-100G-K) and acetone (purity 99.9%, Lot No. MKBR5795V, Sigma W332615-100G), tunicamycin (purity ≥98.0%, Lot No. 037M4047V, Sigma T7765-1 MG).

### 2.2 Exposure of primary human epithelial cells (PBECs) via ALI application

PBECs were isolated from tumor-free resected lung tissue from 4 donors by enzymatic digestion, cells were expanded in keratinocyte serum-free medium (KSFM, Gibco) and stored until usage as previously described in Van Wetering et al. (2000). PBECs were cultured as described in Boei et al. (2017). Briefly, cells were seeded after the first passage on coated transwell tissue culture inserts (Corning Costar, 0.4 µm pore size, 1.12 cm^2^ surface) to grow under submerged conditions for 6 days at 37°C, 5% CO_2_ using a 1:1 mixture of DMEM (Life Technologies, Bleiswijk, the Netherlands) and ‘bronchial epithelial growth medium’ (Lonza, Verviers, Belgium) (B/D medium) with supplementation of BEGM BulletKit singlequots (0.4% [w/v] bovine pituitary extract, 1 mM hydrocortisone, 0.5 μg/ml human hEGF, 0.5 μg/ml epinephrine, 10 μg/ml transferrin and 5 μg/ml insulin, T3 (Lonza) and additional 1 mM HEPES (Lonza), 1 μg/ml BSA (Sigma-Aldrich), 100 U/ml penicillin and 100 μg/ml streptomycin (Lonza) and 15 ng/ml retinoic acid (Sigma-Aldrich). Subsequently, the medium on the cell surface (apical side) was removed to culture the PBECs at the air-liquid interface (ALI), which was maintained up to 28 days at 37°C, 5% CO_2_ with exchange of the ‘bronchial epithelial growth medium’ every 2-3 days followed by apical washing with 100 µL PBS to remove accumulated mucus. Mucociliary epithelial cells differentiation occurs during the maintenance under ALI conditions.

PBECs were exposed to the selected test chemicals under ALI conditions using the P.R.I.T.^®^ ExpoCube^®^ device for 1 h once (acute exposure) or repeatedly on 3 consecutive days (repeated exposure). Supplementary Figure S1 provides an illustration of the exposure setup. Exposures were conducted using a volume flow of 350 ml/min as primary flow for transporting the gas through the exposure device (inline) to the FT-IR monitor. From this primary flow sampling for cell exposure was done using exposure flows of 3 ml/min for each culture. For a detailed description of the P.R.I.T.^®^ ExpoCube^®^ workflow see Ritter and Knebel (2014). Briefly, PBECs cultivated on inserts of a 12-well plate (Corning Costar) included three exposure test lines simultaneously: Exposure to (1) the test chemical, (2) to clean air (negative control) or (3) no exposure (non-exposure control). The test atmosphere was generated by conducting clean air over the surface of the chemical inside a gas washing bottle at 25°C. The resulting atmosphere was diluted with clean air to achieve the desired concentrations for each chemical respectively (see Table 1). The analysis of chemicals during exposure was performed by online measurement using a FT-IR spectroscopy (GASMET, Ansyco, Germany). PBECs were exposed to each chemical in 4-5 concentrations according to pre-experiments. The background of the dose selection is described in detail in supplemental material (SM1). Cellular viability was measured by LDH-leakage and barrier function by measuring the transepithelial electrical resistance (TEER) 24 h after the final exposure (Results in Supplementary Figure S2). At the same timepoint, lysis of PBEC-ALI models for TempO-Seq analysis at BioSpyder was performed. Deviating from this, the non-volatile compound tunicamycin was added to the medium for 24 h, directly followed by lysis. The highest concentration without cytotoxic effect was selected for further omics testing. 500 μL lysis buffer (BioSpyder) was added to the apical side of the PBEC-ALI models, incubated at RT for 10-15 min and transferred to a 96-well plate, this procedure was carried out based on (Yeakley et al., 2017). Samples were stored at −80°C and subsequently shipped on dry ice to BioSpyder technologies (Bioclavis, United Kingdom) for sequencing. All data is from 4 donors as replicates, three of the donors are male and one donor is female.

**TABLE 1:** Chemical exposure concentrations (conc. [ppm]).

Chemical	α-diketone				β-diketone	γ-diketone	Negative substances		Positive substance
	Diacetyl	2,3-Pentanedione	2,3-Hexanedione	3,4-Hexanedione	2,4-Pentanedione	2,5-Hexanedione	Butanone	Acetone	Tunicamycin
**CAS**	431-03-8	600-14-6	3,848-24-6	4437-51-8	123-54-6	110-13-4	78-93-3	67-64-1	11089-65-9
Dose level	Concentration [ppm]								
**1**	102	48 h	49	50	49	50	5000	4997	0,00084495
**2**	135	200	100	206	197	100	10068	9981	0,0084495
**3**	277	506	400	509	990	199	15114	15016	0,02788335
**4**	323	1005	1000^a^	984^a^	3,053	521	20053	20051	0,084495
**5**	1834^a^	5034^a^	4043^a^	5104^a^	4537		25244	25071	0,2788335
**6**									0,84495
**7**									8,4495

^a^ Concentrations that were not included in the further omic analyzes due to their cytotoxicity.

### 2.3 Identification of differentially expressed genes

Transcriptome analyses were carried out with the human TempO-Seq S1500+ assay, which comprises 3,565 genes (Mav et al., 2018). These 3,565 genes were selected by the EUTOXTISK consortium because they are particularly frequently differentially expressed in toxicological experiments, and care was taken to ensure that the selected gene panel covers well-annotated pathways (Mav et al., 2018).

The RNA sequences per probe were mapped against the human ensemble transcriptome (hg38 aka GRCh38v100) to obtain ensemble gene IDs.

Counts were normalized by counts per million. Within quality control, samples with low read counts corresponding to a library size <500 k and genes with an overall variance of 1 were discarded. The controls were grouped by time point and compound. A batch correction was carried out according to gender. The differentially gene expression analysis was done with DESeq2 in R (version 1.32.0) (Love et al., 2014), considering an adjusted *p*-value of <0.05 (Benjamini-Hochberg method) and an absolute log2FoldChange of 1 or higher.

### 2.4 Pathway analysis

A pathway analysis was carried out taking into account the six databases from ConsensusPathDB (Version 35, Barel and Herwig, 2018). 2,603 signaling pathways were considered, these signaling pathways included at least four genes of the TempO-Seq S1500+ gene panel.

The preselection of the most responsive genes interferes with pathway enrichment analysis, that is normally performed, as it uses statistical significance tests such as the Fisher exact test to distinguish a random finding from a specific activation (García-Campos et al., 2015).

In this study, we therefore used an approach recently described by (Barel and Herwig, 2018). Differentially expressed genes (g) were weighted for the analysis of pathways by calculating a gene score (S) for each condition (c). A condition is the combination of time point, compound, and concentration. Gene scores take into account the significance (adjusted *p*-value) and the observed absolute log2fold change of the differentially expressed genes.

$$S_{gc}=abs\left(\log2FoldChange\right)*abs\left(\log10\left(p-value\right)\right)$$

Thereafter, a pathway score (M) for each pathway (l) was calculated for each condition (c) using the gene scores. For this purpose, the scores of all DEGs are summed and divided by the number of genes (m) included in the pathway and measured in the S1500+-panel.

$$M_{cl}=\frac{1}{m}\underset{gi\in M}{\sum }S_{gc}$$

In order to be able to compare the results obtained, a normalization over all pathways for a condition was carried out (RPR).

$$RPR_{l}=log2\left(M_{cl}/median\left(M_{c}\right)\right)$$

Pathways with a RPR greater than two were considered as activated.

### 2.5 Upstream analysis

Upstream Analysis is an integrated promoter–pathway analysis.

Upstream analyses were performed on gene sets of 300 differentially expressed genes per time point and substance and group using the geneXplain platform (Stegmaier et al., 2017).

In addition, a group specific DEG profile was developed for the α-diketones. To determine the most representative genes being affected by α-diketone exposure, DEGs commonly observed following exposure to all α-diketones (in at least one concentration group tested) were selected. For the substance profiles, the top 300 DEGs were selected based on the average absolute highest log2 fold change.

To obtain information about potentially involved transcription factors, promoters of the differentially regulated genes are retrieved and analyzed for potential transcription factor (TF) binding sites in a first step. From these binding sites, a set of postulated TFs is identified that potentially have regulated the found DEGs.

The search for enriched transcription factor binding sites (TFBSs) from promoters of DEGs per substance, time point and concentration and for the groups and substance profiles was performed using the geneXplain platform (Kolpakov et al., 2011) and the corresponding ‘Identify enriched motifs in promoters (TRANSFAC^®^)’ workflow (Koschmann et al., 2015). Gene promoters with a range from −1000bp to +100bp from transcriptional start sites were screened for potential TFBSs based on known motifs in the TRANSFAC^®^ database, which is a collection of positional weight matrices (PWMs) of binding sites and corresponding transcription factors. A total number of 394 matrices were used whose associated transcription factors are known to be expressed in lung tissue (TRANSFAC^®^ database version 2021.1. The algorithm estimates an enrichment analysis for binding sites that occur more frequently compared to a background set, which was a random set of 900 genes and corresponding promoters that are expressed in human lung tissue (source: HumanPSD database). The result is a list of the binding and the associated set of human transcription factors that are likely responsible for the differential regulation of the observed set of DEGs.

Starting from this, a further step of upstream analysis was performed to search for master regulators (MR) that are known to activate the identified potential TFs. Master regulators are viewed as those regulation units showing a potential key functionality of several underlying biological and toxicological processes. A molecular network analysis tool called “regulator search” was used to identify MRs (Kel et al., 2006). The principle of the regulator search can be briefly summarized as follows: the TRANSPATH database (version 2021.1) comprises a large directed protein network, where the nodes are proteins and the edges are reactions in different pathways. The postulated TFs are represented as nodes in this network.

As mentioned above the analysis started from the identified TFs, the algorithm searches for up to 10 upstream nodes and postulates MRs, which represent highly interconnected proteins (by a score >0.2) and specific proteins for the DEG input set (by a z score >1) (Kel et al., 2006).

### 2.6 Reconstructed signaling protein networks

The reconstructed networks were developed to visualize a connection to the Mechanism of Action and the MRs. The reconstructed networks connect genes that could be assigned to a specific mode of action and the MRs were then mapped onto this network in order to obtain comparability between the MRs of the different group/substance profiles.

Protein lists were obtained by multiple queries from different databases such as HumanPSD™ KEGG, Wikipathways (Wingender et al., 2007), which contain disease and signaling network information observed in humans for three adverse outcomes, namely, “pulmonary fibrosis,” “inflammation” and “apoptosis.” With the obtained three protein lists a clustering algorithm within the geneXplain platform was used to reconstruct signaling protein networks based on evidence of protein-protein interactions from the TRANSPATH^®^ database (Krull et al., 2006). In the TRANSPATH^®^ database protein-protein interactions occurring in humans manual curated from peer-reviewed publications and assigned evidence levels and reliability scores for protein reactions obtained from experiments for proteins existing *in vivo* are represented. Inside the procedure for a reconstruction of protein networks we allowed a maximum of three predicted protein reactions apart from the input protein list. Cytoscape (version 3.8.2) was used for the visualization of all reconstructed protein networks (Kohl et al., 2011).

## 3 Results

### 3.1 Substance characterization

**FIGURE 1:**
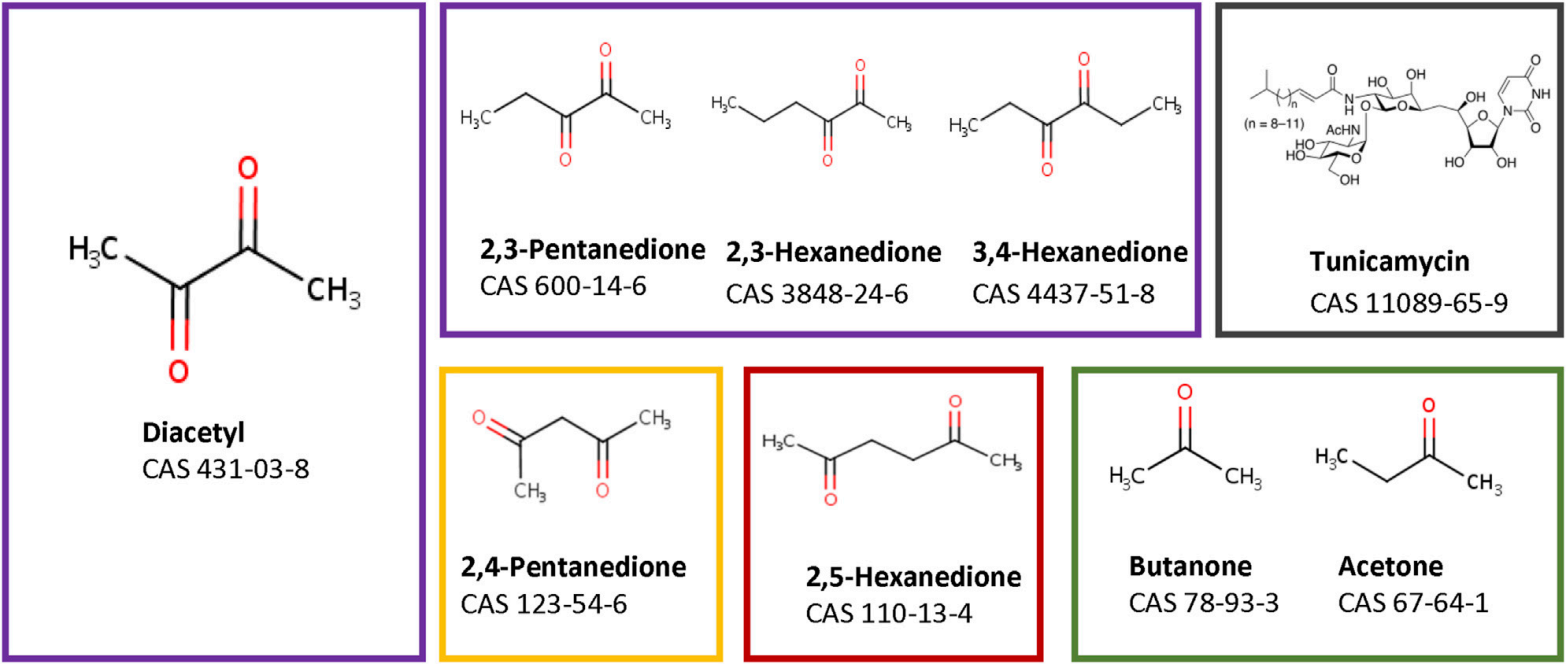
Chemical structures of the tested substances. Purple edges the α-diketones, orange the β-diketones, red edges the γ-diketones, the negative substances are edged in green, and tunicamycin as a substance with a different MoA has a black edge.

The read-across case study investigates nine compounds, which are grouped into five groups based on their structural as well as toxicological properties (Figure 1, grouping indicated by coloured boxes). One group contains four α-diketones, whose structure differs in the length of the hydrocarbon side chains (purple box). The α-diketone Diacetyl is known to cause bronchiolitis obliterans in employees who made butter-flavored microwave popcorn. Its mechanism of action has been well studied *in vivo* (Zaccone et al., 2013). It is also known from preclinical *in vivo* studies in rats that the two other α-diketone 2,3-pentanedione and 2,3-hexanedione have the same mode of action (Zaccone et al., 2013). The observed progression of toxicological findings of both compounds proceeds from injuries to the epithelial tissue via immune processes to bronchial epithelial scarring (Gwinn et al., 2017). The toxicological properties of 3,4-hexanedione are largely unknown. 3,4-Hexanedione was tested in a subchronic study in rats wherein no effect was observed up to a single oral dose of 17 mg/kg bw/day (Posternak et al., 1969). An inhalation study is not available.

Two structurally slightly different diketone compounds, 2,4-pentanedione (β-diketone, yellow box) and 2,5-hexanedione (γ-diketone, red box) were also included into the assessment. 2,4-Pentanedione causes inflammation in the upper airways of rats (DODD et al., 1986) whereas 2,5-hexanedione induce peripheral neuropathy after subcutaneous injection (Spencer and Schaumburg, 1975; Ichihara et al., 2019).

In addition, two ketones, acetone and butanone, were selected as negative substances (green box), as both substances did not provoke any adverse effect after repeated inhalation exposure in preclinical studies (Bruckner, 1981; Cavender et al., 1983).

Also the structurally very different compound tunicamycin was chosen because of its well-known mode of action, which is not related to pulmonary inflammation and fibrosis. Tunicamycin blocks protein folding of glycoproteins by inhibiting N-glycosylation (Wang et al., 2015), resulting in induction of the unfolded protein response. This is under the control of three sensors, namely, PERK, IRE1a and ATF6, each activating different signaling cascades (Yang et al., 2020).

### 3.2 Concentration dependency and impact of exposure duration on transcriptome data and pathways analysis

**FIGURE 2:**
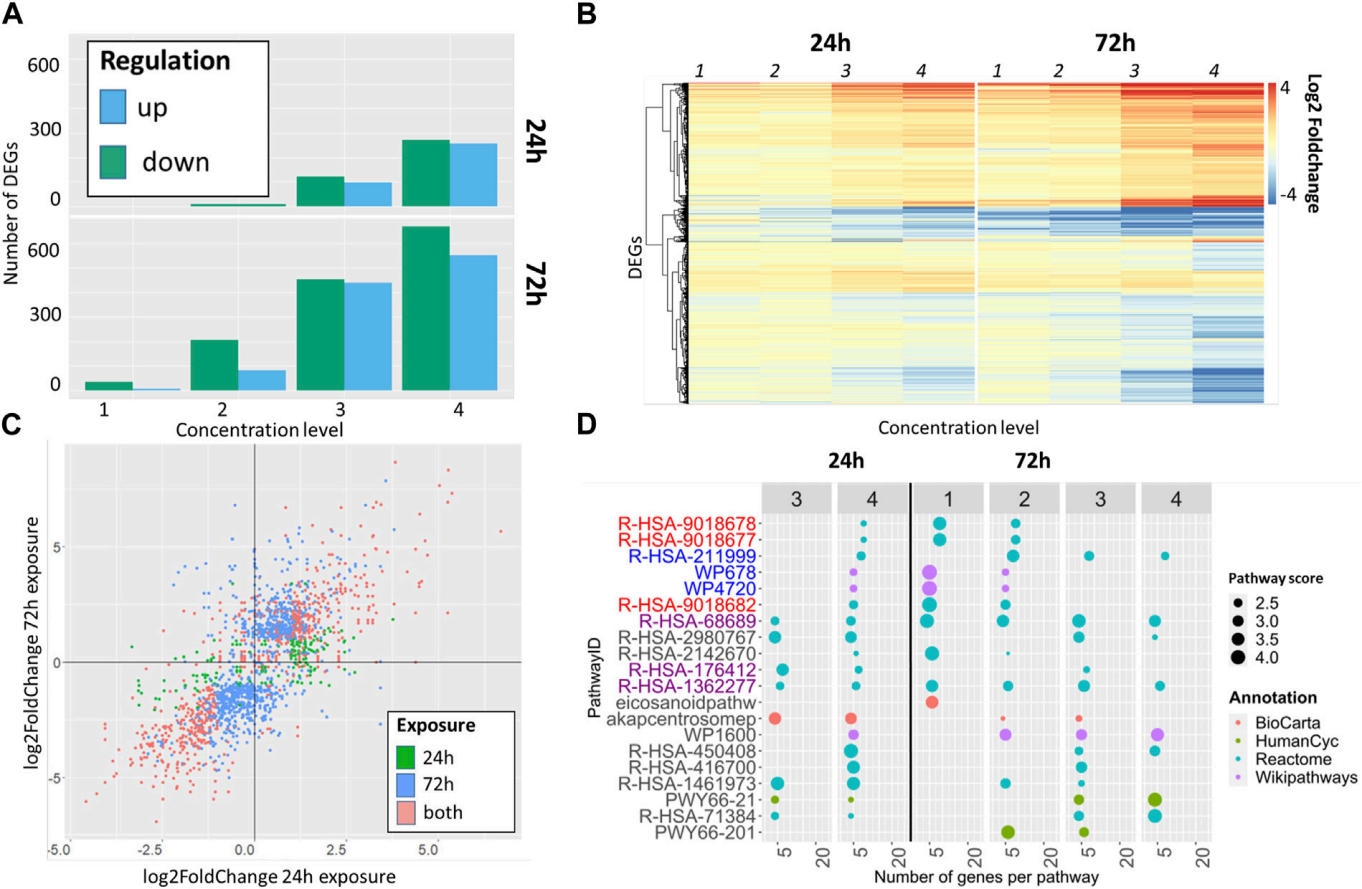
Comparison of DEG and pathway enrichment analyses for diacetyl after single (24 h) and repeated exposure (72 h): **(A)** Number of DEGs for diacetyl classified by upregulated and downregulated genes. **(B)** heatmap of DEGS showing similarities of log2foldchanges between single and repeated exposure. **(C)** Correlation of log2fold changes of DEGs at the highest tested concentration (concentration level 4), the colour schema indicates differentially expressed genes obtained after single (green), repeated (blue) or in both approaches (red). **(D)** Comparison of 10 pathways per time point with highest pathways scores. Pathways are colored according to their known mode of action: red—inflammation, blue - drug metabolism, purple - fibrosis, black—other.

The impact of exposure time on the resulting transcriptome data was evaluated using the α-diketone Diacetyl as an example. In parallel the concentration dependent effects of diacetyl on the transcriptome were analyzed. Overall, Diacetyl shows an increasing number of DEGs with increasing concentration levels (Figure 2A). Acute exposure led to 10 downregulated DEGs at the lowest concentration. At the highest concentration 257 up- and 271 downregulated DEGs could be shown whereas repeated application over 3 days resulted overall in more DEGs, with 551 up- and 668 downregulated genes at the highest concentration (Figure 2A).

The heat map shows that the DEGs have a common expression pattern, i.e., they are up- or downregulated together, both within the single and repeated exposure, as well as considering the two exposure times. The absolute log2FoldChange of the observed DEGs revealed a concentration dependent increase (Figure 2B).

DEGs obtained from acute and repeated exposure show a high concordance with regard to their up- and downregulation (Figure 2C, colored in red). The same trend is observed for the majority of differentially expressed genes after repeated exposure (colored in blue) and for the few DEGs from acute exposure (Figure 2C, colored in green).

The pathway enrichment analysis resulted in a total of 296 pathways for diacetyl. 173 pathways were obtained after acute exposure, 212 after repeated exposure, with 89 shared pathways for both time points. The ten top regulated pathways, defined by the highest pathways score, were compared in more detail per time point. There are no overlaps between the pathways for these top 10 pathways per time point, but all pathways that were already active in the case of a single exposure also appear in the case of repeated exposure, and only two pathways are not active in the case of the single exposure. Despite this high qualitative concordance, repeated exposure tends to induce more pathways at overall lower concentrations compared to acute exposure conditions.

**FIGURE 3:**
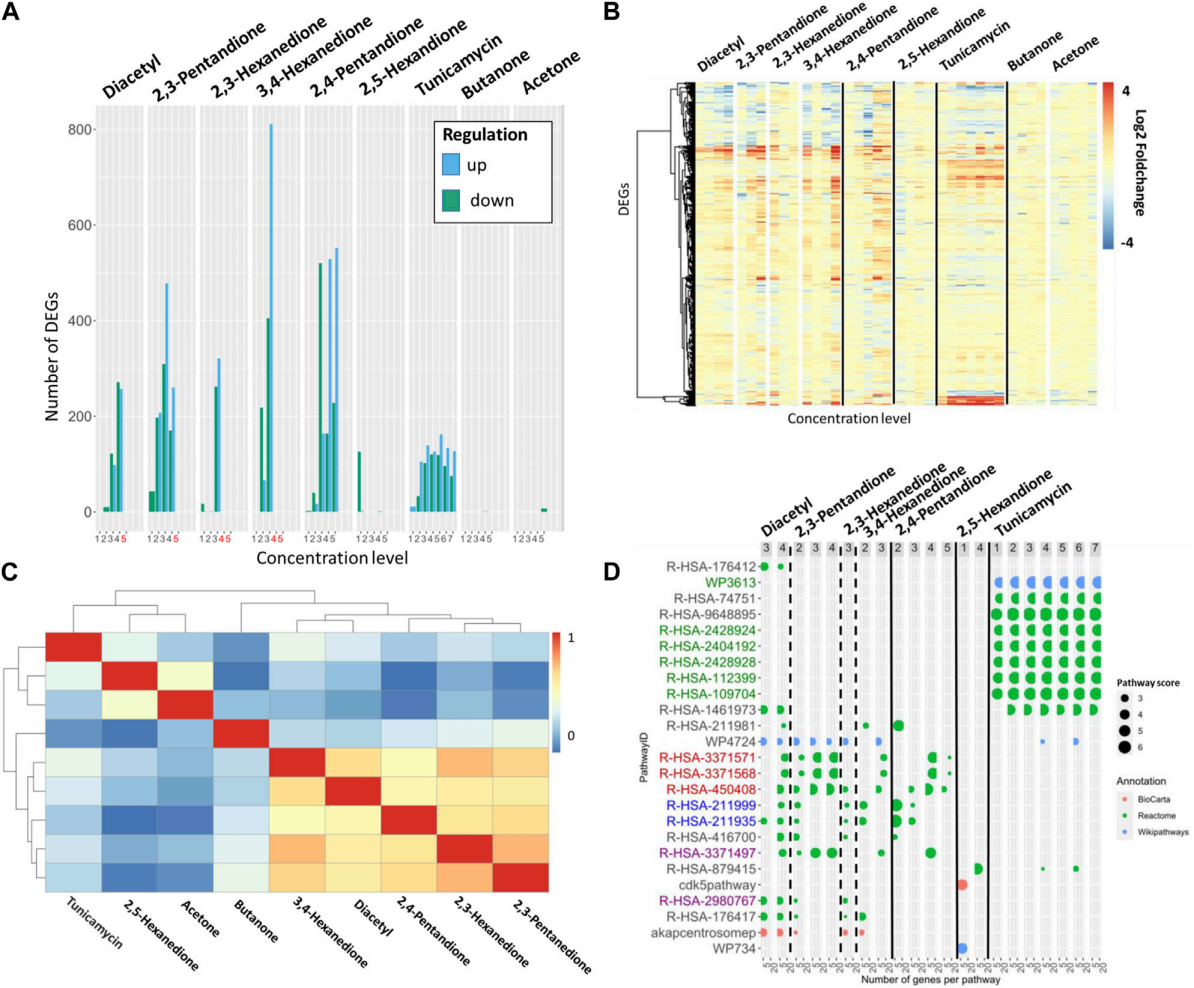
**(A)** Number of DEGs per substance and concentration. Dose groups with a cytotoxicity above 20% are indicated in red. All compounds were tested using 5 dose levels, tunicamycin was tested with 7 dose levels. **(B)** Log2FoldChange of all genes that are DEG at least once per substance in increasing concentration. The genes are arranged with hierarchical clustering. **(C)** Correlation matrix. The correlation scores ordered with hierarchical clustering were determined using the Spearman coefficient of the log2FoldChange-values. **(D)** 30 pathways are shown, which are derived from the ten pathways with highest pathway scores for diacetyl, α- and β-diketones and tunicamycin. Pathways listed in the table are colored according to their mode of action, red for inflammation, blue for drug metabolism, green for protein folding and purple for fibrosis.

Pathways are colored according to their MoA (Figure 3D; Table 1). All three pathways associated with fibrosis are observed after single and repeated exposure (Figure 2D, purple).

These results indicate that under the exposure conditions chosen in this study, the exposure time has an impact on the transcriptome response. Repeated exposure led to more pronounced responses regarding the number and the fold change of differentially expressed genes and the thereof resulting number of enriched pathways. Further a trend towards responses at lower dosing was observed.

Both single and repeated exposure lead however to inflammation- and fibrosis-related signalling pathways relevant to the presumed mode of action of diacetyl.

All other compounds in this study were tested under acute exposure conditions for the inter- and intra-group comparisons in order to keep the experimental approach as simple as possible, but as complex as necessary.

### 3.3 Inter- and intra-group comparison

As observed for diacetyl, the other diketone compounds in this case study also show dose-dependent responses at the transcriptome level, indicated by an increase in the number of differentially expressed genes and the absolute increase in log2FoldChange (Figures 3A, B).

The highest concentration doses tested for diacetyl and the two highest doses tested for 2,3-hexanedione and 3,4-hexanedione resulted in greater than twenty percent cell death. Therefore, these cytotoxic concentrations were excluded from subsequent transcriptome analyzes as it is expected that they predominantly exhibit DEGs and signaling pathways associated with apoptosis and necrosis. This phenomenon is known as a cytotoxic burst (Judson et al., 2016) and it is assumed that it does not provide relevant information about the compound-specific mechanism of action of the investigated compounds.

Within the α-diketones, a high correlation of up- or downregulated DEGs is observed, as indicated by a Spearman’s coefficient above 0.6 (Figure 3C). The β-diketone 2,4-pentanedione shows a very similar DEG profile compared to the α-diketones such as, e.g., visualized in the heat map of DEGs (Figure 3B) or the overall high Spearman coefficient of >0.5 compare to the α-diketones (Figure 3C); while the γ-diketone 2,5-hexanedione remains inactive up to the highest dose tested *in vitro*. In line with the absence of toxicity findings in vivo studies, the two negative control compounds, butanone and acetone, are inactive and do not induce DEGs.

Tunicamycin induces a total of 417 differentially expressed genes in a dose dependent manner. With increasing concentrations an increasing number of DEGs and also increasing absolute log2FoldChanges per DEG are observed. The DEGs expression pattern of tunicamycin differs remarkably from the one observed for the α- and β-diketones (Figure 3C; Spearman correlation score <0.4). 144 DEGs of tunicamycin are not differentially expressed in the α- and β-diketones, whereas 137 DEGS are differentially expressed in both analyses but with opposite log2FoldChange (Figures 3A–C). 230 DEGs, almost half of the DEGs of tunicamycin, are in common with the DEGs seen for α- and β-diketones and show similar up and downregulation.

The total number of pathways for the active diketones includes 861 different pathways and for tunicamycin 293 pathways. Again, the pathways with highest pathway scores are compared between α-, β-diketones, diacetyl and tunicamycin to better understand similarities and differences in biological processes. A literature review was performed to assign a mode of action to the best pathways (Table 2 showing these 10 top regulated pathways).

**TABLE 2:** Pathways to which a mode of action could be assigned.

Pathway ID: Pathway name^a^	Compound-groups	Mode of action	Source
R-HSA-3371571: HSF1-dependent transactivation, R-HSA-3371568: Attenuation phase, R-HSA-450408: AUF1 (hnRNP D0) binds and destabilizes mRNA	α- and β-diketones	Inflammation	Quintana and Cohen (2005)
R-HSA-211999: CYP2E1 reactions, R-HSA-211935: Fatty acids	α- and β-diketones	Drug metabolism	Guengerich (2006); Gonzalez (2005)
WP3613: Photodynamic therapy-induced unfolded protein response, R-HAS-2404192: Signaling by Type 1 Insulin-like Growth Factor 1 Receptor (IGF1R), R-HSA-2428924: IGF1R signaling cascade, R-HSA-112399, R-HSA-109704:PI3K Cascade	Tunicamycin	Protein folding (Regulation with IGF1 and PERK)	Weijer et al. (2015); Yang et al. (2020)
R-HSA-331497, R-HSA-2980767: Activation of NIMA Kinases NEK9, NEK6, NEK7	α-diketones	Fibrosis	Korthagen et al. (2012)

^a^ IDs come from the databases merged into the ConsensusPathDB, in this case Reactome and Wikipathways.

Most pathways associate with α-diketones occur in at least one concentration level in each substance, including pathways that have been linked to inflammation, fibrosis, and drug metabolism (Table 2). The fibrosis pathways occur within all α-diketone substances, except for 3,4-hexanedione. A possible explanation for the absence of fibrotic pathways in the case of 3,4-hexanedione is that only one subcytotoxic concentration contributed to the analysis.

The β-diketone 2,4-pentanedione shares 119 out of 688 (union) pathways with the α-diketones, but derives overall a lower number of active pathways (308 pathways, Figure 2D; Table 2). Also in this case, the fibrosis pathways were not identified within the top regulated pathways.

Of the top ten pathways for tunicamycin, seven could be associated with protein folding, consistent with the known mechanism of action. There is only one pathway that overlaps with the diketone (R-HAS-1461973) and it is related to immune responses (Bevins, 2006).

### 3.4 Upstream analysis

An upstream analysis for individual compounds and the group of α-diketones investigated the regulation of the observed DEGs via transcription factors (TFs) and master regulators (MRs) (Table 3).

**TABLE 3:** Number of transcription factors (TFs) and master regulators (MRs) for different gene profiles; all-total number of identified TFs/MRs; specific - number remaining when TFs/MRs after exclusion of TFs/MRs detected for tunicamycin, acetone and butanone.

Profile	TF (all/specific)	MR (all/specific)
α-diketones	30/1	164/25
Diacetyl	33/2	242/64
2,3-Hexandione	20/0	142/65
2,3-Pentanedione	22/1	221/63
3,4-Hexandione	21/1	111/38
2,4-Pentanedione	2/0	142/26

Significant binding site enrichment led to comparable numbers of postulated TFs, ranging from 33 TFs for diacetyl to 20 TFs for 2,3-hexanedione (Table 3, TF (all)); whereas the number of MRs differ a bit more ranging from 242 master regulators for diacetyl to 111 for 3,4-Hexandione (Table 3; MR (all)). In order to obtain more specific TFs and MRs per group/compound, the TFs and MRs also obtained from the negative control compounds acetone and butanone were not taken into account. Also, TFs and MRs shared with tunicamycin, which has a different mode of action, were not considered. For the α-diketones, the number of specific TFs and MRs decreased to 5 and 33, respectively. Diacetyl showed most specific TFs (N = 2) and MRs (N = 64) (Table 3). Overall, a total of three different specific transcription factors were identified (Table 3).

All three specific postulated TFs are known to be expressed in the human lung according to protein atlas version 21.0 (Table 4). All specific transcription factors were found in profiles for the alpha-diketones. Two are associated with pulmonary fibrosis (NKX2-1 and GATA6), another is involved in various cellular stress signaling pathways (ATF5).

**TABLE 4:** The specific transcription factors with expression in the lung, the profiles in which they occurred and the mode of action to which they could be assigned.

ID	name	Expression in lung^a^	Profiles	Mode of action	Source
ENSG00000136352	NKX2-1	yes	α-diketone group, Diacetyl, 2,3- Pentanedione	Lung fibrosis	Borie et al. (2021)
ENSG00000169136	ATF5	yes	Diacetyl	Cellular stress	Zhou et al. (2008)
ENSG00000141448	GATA6	yes	3,4-Hexanedione	Lung fibrosis	Leppäranta et al. (2010)

^a^ Information taken from the Human Protein Atlas (https://www.proteinatlas.org).

Over all profiles 145 specific master regulators were found. Due to this high number, it was analyzed whether regulators for the well-known mode of action can be found within the α-diketones profile by considering master regulators that appeared in at least four gene profiles and appeared in the α group profile or diacetyl compound profile (Table 5). Of these six master regulators, three master regulators are found to be associated with pulmonary fibrosis (ERBB2, EGFR and FGFR2) and one with inflammation (FYN). All three master regulators associated with lung fibrosis are also included in the β-diketone compound profile.

**TABLE 5:** Master regulators present in at least four gene profiles and present in the diacetyl compound profile or the α group profile.

ID	name	Expression in lung^a^	Profiles	Mode of action	Source
ENSG00000168621	GDNF	Yes	α, Diacetyl, 2,3-Hexanedione, 2,3-Pentanedione, 3,4-Hexanedione, 2,4-pentanedione	No data	No data
ENSG00000141736	ERBB2	Yes		Lung fibrosis	Schramm et al. (2022)
ENSG00000146648	EGFR	yes	α, Diacetyl, 2,3-Hexanedione, 2,3-Pentanedione, 3,4-Hexanedione, 2,4-pentanedione	Lung fibrosis	Schramm et al. (2022)
ENSG00000276023	DUSP14	yes		Tuberculosis	Hijikata et al. (2016)
ENSG00000010810	FYN	yes	All compound profiles	Inflammation	Mkaddem et al. (2017)
ENSG00000197122	SRC	yes	Diacetyl, 2,3-Hexanedione, 2,3-Pentanedione, 3,4-Hexanedione, 2,4-pentanedione, 2,5-Hexandedione	Lung fibrosis	Li et al. (2020)

^a^ Information taken from the Human Protein Atlas (https://www.proteinatlas.org).

All diketone profiles examined show that the most common specific MRs support a common mechanism of action, and this is the well-known MoA.

### 3.5 Reconstructed networks

Another challenge in the read across assessment is the visualization of similarities with regard to the observed mode of actions.

In order to address this question, reconstructed protein networks were developed for three different biological processes, namely, lung fibrosis, inflammation, and apoptosis. Each node in the network represents a protein which plays a role in the three MoAs. These annotations are based on manually curated data from human stored in the Transpath database.

**FIGURE 4:**
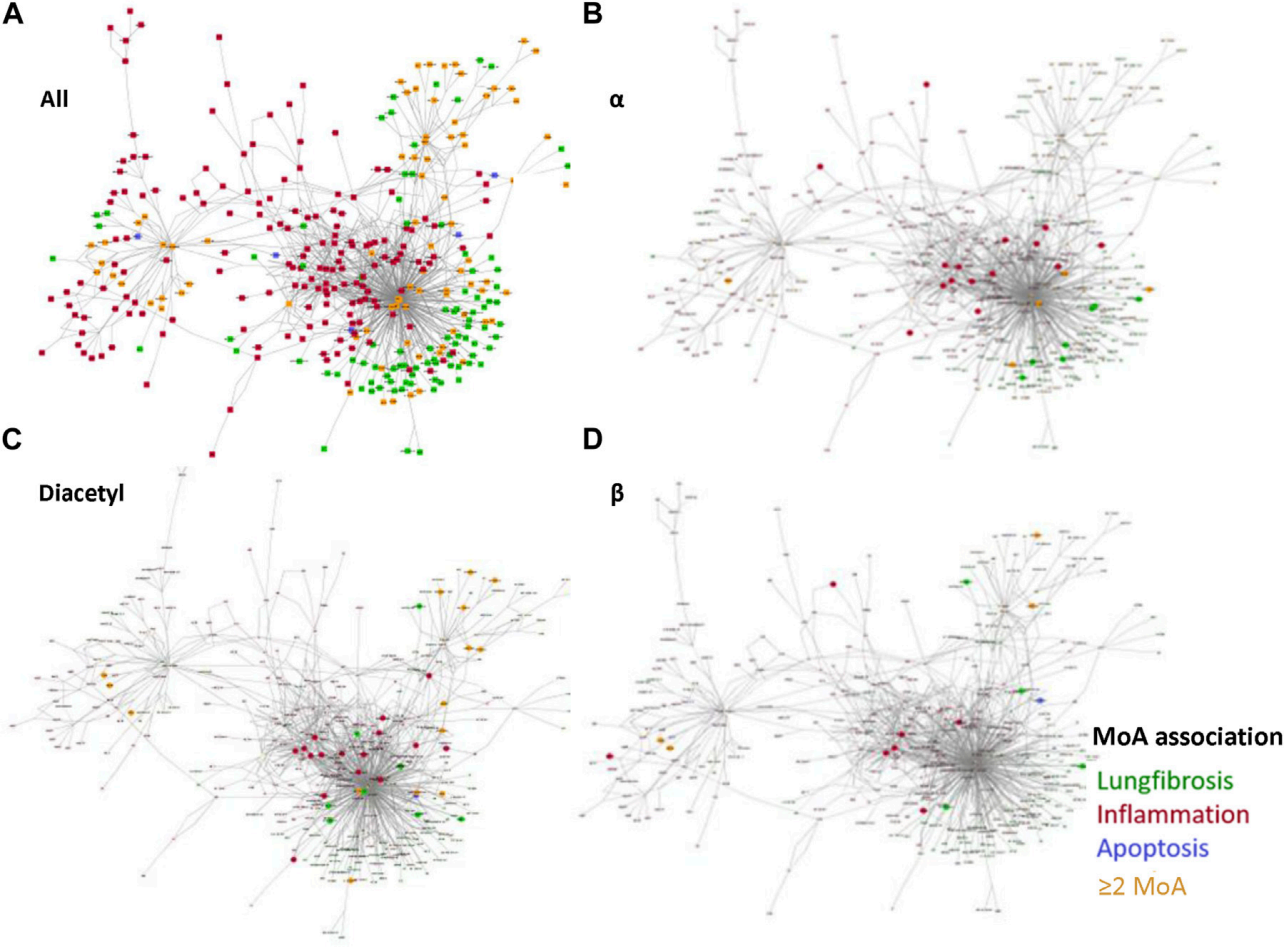
Reconstructed networks to visualize the obtained MRs in this read-across case study. **(A)** All MRs related to pulmonary fibrosis (green); inflammation (red) and apoptosis (blue). Nodes belonging to more than one of the three MoAs are colored in yellow. The same color code is applied to visualize the MoAs of three different profiles: **(B)** MRs identified in the α-diketone group, **(C)** MRs identified for the α-diketone diacetyl, and **(D)** MRs identified for the β-diketone 2,4-pentanedione.

213 nodes are obtained specifically for pulmonary fibrosis, 429 for inflammation and 39 for apoptosis. Other nodes belong to more than one MoA. Seven nodes are present in all three biological processes, whereas 42 nodes intersect between lung fibrosis and apoptosis and seven nodes overlap between inflammation and lung fibrosis (Figure 4).

To visualize common MoAs per group and compound profile, the master regulators were mapped to the nodes of the network.

The master regulators of the alpha group profile map primarily to the nodes for inflammatory processes, but also to multiple nodes associated with pulmonary fibrosis (Figure 4B).

Diacetyl is shown as one example representing the outcomes of the four different alpha diketone profiles. Diacetyl show a large number of nodes associated to inflammation and also some associated to pulmonary fibrosis (Figure 4C). Similar results were obtained for the three other alpha compound profiles (data not shown). Compared to the alpha-diketone profiles, the beta-diketone profile shows a smaller number of nodes, which are primarily associated inflammatory processes (Figure 4D).

## 4 Discussion

Even though RAx is a well-known and often used approach in regulatory chemical risk assessment to fill a data gap and to avoid animal testing (ECHA, 2020), it is often not accepted by regulatory agencies. One reason for this is a lack of evidence for common toxicodynamic properties within the grouped compounds, as *in vivo* studies reveal apical findings whose underlying mechanisms are often unknown (Ball et al., 2016).

There is therefore a need to integrate mechanistic data into read-across assessments, to provide evidence that the grouped compounds will cause similar toxic effects in the human organism or a consistent trend. An open question is how mechanistic data from omics approaches such as transcriptome data, can support the assessment of a similar mode of action.

Similarity assessment is highly dependent on the problem formulation. Similarity for genotoxic compounds will need other evidence compared to compounds which inhibit specific enzymes, like organophosphates inhibiting cholesterinesterase. The main question is therefore, to what extent are compounds similar in relation to which endpoint?

Similarity scores can be calculated in many different ways. This is a well-known phenomenon, e.g., from chemical similarity assessments, where binary data on the presence or absence of unique and common molecular features are used to calculate scores with simple algorithms such as Tanimoto or Dice (Willett et al., 1998). A similar simplistic approach could be used for transcriptome data comparing the absence of presence of unique and shared DEGs, pathways or regulated proteins/master regulators. Similarity scores alone do, however, not evaluate the underlying mechanisms and can therefore be probably considered as starting point of the assessment assuming that similar gene profiles indicate a regulation of similar biological processes. Low similarity on the gene level, does however, not directly indicate a dissimilar mode of action, as a pathway enrichment or upstream analysis might reveal that same biological processes regulated. Transcriptome data need thus other analyses strategies compared to similarity assessment of physico-chemical parameters.

This case study provides further insights into the assessment of mode of actions and similarity within grouped compounds by using transcriptome data. We believe that case studies such as this are necessary to develop analysis strategies and acceptance criteria, in particular the level of evidence considered sufficient to establish similarity.

To date, it is not known, which cellular assay is most appropriate to investigate mechanistic data for read-across assessments. The recently published EUTOXRISK read-across strategy recommends that the *in vivo* data and potentially available AOPs shall guide and inform the NAM testing strategy (Escher et al., 2019). For this case study on compounds that might induce lung fibrosis, primary human lung epithelial cells were chosen, as they are relatively close to the human *in vivo* situation. A comparison to other test systems was not carried out, but it is likely that other models like 3D cell, *ex vivo* or co-culture models could also give valuable information. A systematic comparison of test systems differing with regard to complexity could help to distinguish between problems in which a relatively simple cell test already provides sufficient evidence and those in which more complex test systems are required. This was however, not in the scope of the current case study.

In our case study, the group of four α-diketones show a highly similar expression pattern of DEGs. A large number of shared genes were upregulated or downregulated after both single and for diacetyl also after repeated exposure and across different concentrations. This DEG pattern clearly differed from those of tunicamycin, as shown by Spearman correlation, in agreement with its different mode of action. These data confirm the recent described hypothesis that gene expression data, even from short term *in vitro* assays, can serve as first indication of similar biological properties (Escher et al., 2019; Vrijenhoek et al., 2022). However, despite their completely different MoA, tunicamycin and the a-diketones has about 230 genes in common. It can be hypothesized that these DEGs represent to a large extent adaptive cellular responses to any kind of chemical stressor. This hypothesis has to be further investigated by testing compounds with different MoAs and could help to distinct unspecific from MoA-specific cellular responses.

The β-diketone caused a very similar gene expression pattern, thus indicating that this compound has a very similar mode of action as compared to the α-diketones. This finding is in good agreement with the available data from preclinical studies, in which the β-diketone also induced pulmonary fibrosis.

The γ-diketone and the two negative compounds were inactive up to the highest *in vitro* tested concentration. An analysis of biological similarity is therefore not possible based on transcriptome data. Two hypotheses can be made to explain the observed inactivity, the compounds might either be less potent compared to the other diketones or has another mode of action. The latter seems to be most probable for the γ-diketone, which induces neurotoxic effects after oral exposure in rodent (Dorry et al., 2020) studies (Ichihara et al., 2019). The transcriptome data did however not help to elucidate the difference. A statement on a similar or dissimilar mechanism of action cannot be made based on inactivity, a finding that was also recently reported by Vrijenhoek et al. (2022).

Transcriptome data can also be used to investigate potency difference between the grouped compounds by analyzing benchmark concentrations per DEG or signaling pathway (Phillips et al., 2019). In this case study a quantitative assessment of potency differences was not possible, mainly because of the non-optimal dose selection. Some of the tested concentrations already induced cytotoxicity and the number of remaining available concentrations were insufficient for benchmark modelling. This aspect needs to be further addressed in future investigations.

A pathway and an upstream analysis were carried out to investigate the biological processes associated with the observed DEGs. The pathway analyses, just like the DEG analysis, revealed common most active pathways between the alpha- and beta-diketones and a distinct demarcation from the pathways to tunicamycin. The S1500+ panel measured in this read-across case study contains a relatively small number of genes, which are selected because of their particularly responsiveness. A classic signaling pathway enrichment analysis was therefore not possible due to the relatively high number of DEGs compared to the number of genes measured. One way to deal with this problem is to extrapolate the gene set to the entire transcriptome (Mav et al., 2020) or set thresholds to set a minimum number of DEGs per pathway (Ramaiahgari et al., 2019). The latter approach was followed in this case study and was also successfully applied in the NTP and ToxCast project (Judson et al., 2016). In this work we calculated a pathway score based on the values for the differentially expressed genes. The advantage of the method we have chosen is that with the log2FoldChange and the *p*-value a qualitative evaluation was included, since the pathways with a particularly high average log2FoldChange and a small average *p*-value receive a higher pathway score and thus a higher value. As part of the upstream analysis and the subsequent visualization with the reconstructed networks, many common postulated TFs and MRs were found for the α− and β−diketones as well as tunicamycin compound. The identified TFs and MRs relate to many basic cell functions, including cell proliferation and stress responses, in addition to specific responses leading to the mechanism of action. To distinguish potentially non-specific adaptive cellular responses from more specific effect responses, a filtering step was applied in which TFs and MRs observed under multiple conditions and test compounds were excluded. Using this approach, a common mode of action within the α- and β-diketones was identified and distinctions to the tunicamycin compound could also be made.

Further examples, exploring, e.g., groups of compounds with more diverse mode of actions are needed to generalize this approach and to enhance the understanding about the specific and potentially adaptive TFs and MRs.

Also in the upstream analysis, the relatively small gene set of the S1500+ panel was a disadvantage, since potential postulated TFs and MRs that indicate the mode of action might have been missed.

Six AOPs on pulmonary fibrosis are currently described in the AOP-Wiki. These pathways all have in common that binding to different types of receptors lead to pulmonary fibrosis via inflammation and collagen accumulation (Society for the Advancement of AOPs, [2022]). Proteins associated with inflammation were identified in the reconstructed networks after a single exposure to the α- and β-diketones and are consistent with the AOPs. The reconstructed networks appear to be a useful tool to visualize mechanistic similarities and to infer an adverse outcome such as fibrosis based on early processes in vitro cell cultures. All active alpha- and beta-diketone substances show master regulators that can be assigned to pulmonary fibrosis and inflammation, which corresponds to their known mechanism of action. It is however noted that many nodes are inactive in the reconstructed networks. One possible explanation is that the short-term *in vitro* assays used in this case study do not show the full development process of fibrosis, like, e.g., collagen formation (Lynch, 2009). In addition, many different signaling pathways are known to lead to inflammation and fibrosis, such as EGFR signaling pathway, TORC2 pathway and TGFβ pathway (Chang et al., 2014; Schramm et al., 2022)) which do not all have to be relevant for diketones. One limitation of the reconstructed networks is, that they currently do not include information about the relationship between the obtained nodes such as inhibition or activation. Further work has to be done to include these interactions, to allow a more detailed mechanistic interpretation and to better discriminate between the biological processes.

## 5 Conclusion

Read-across assessments are always based on a read-across hypothesis, which is derived from the available *in vivo* data of data rich source compounds. For the purpose of this case study, we can assume that only diacetyl is a data rich source compound, and its toxicity data after repeated exposure shall be used to predict the structurally similar α,β, and γ-diketones. The read-across hypothesis is thus, that all structurally similar compounds will cause pulmonary fibrosis.

Overall, there is high concordance between the *in vivo* and *in vitro* results for the source compound diacetyl. This finding is necessary to gain confidence that the *in vitro* approach is able to address the read-across hypothesis.

This case study shows that transcriptome data can be used to indicate a common mechanism of action for compounds that are active at the level of gene expression. Inactive compounds cannot be evaluated. We have shown that there are similarities within the alpha and beta diketones at the level of gene expression and also in the biological interpretation. It was helpful to include tunicamycin, a compound with different mode of action, as it allows to discriminate better the differences in the obtained gene and pathway profiles.

The investigation of transcriptome data and their inference of an adverse outcome can thus substantiate the assessment of similar toxicodynamic properties with regard to a read-across hypothesis. We therefore believe that this approach can significantly reduce the uncertainty within read-across assessments and by this increase its acceptability. It has however, to be investigated further which level of similarity would be considered “acceptable” e.g., by regulatory bodies.

## Data Availability

The datasets presented in this study can be found in online repositories. The names of the repository/repositories and accession number(s) can be found below: https://www.ebi.ac.uk/biostudies/eu-toxrisk/studies?facet.eutoxrisk.project_part=cs8, S-TOXR1829, S-TOXR1814, S-TOXR1824, S-TOXR1825, S-TOXR1826, S-TOXR1827.

## Abbreviations

DEGs, differentially expressed genes; ECHA, European Chemical Agency; EFSA, European Food Safety Authority; GLP, good laboratory practice; hOED, human oral equivalent dose; MoA, mode of action; NAM, new approach methodology; RAx, read across.
