# Supplementary material for: Substantiate a read-across hypothesis by using transcriptome data—A case study on volatile diketones
Source: Front Toxicol. 2023 May 3;5:1155645. doi: 10.3389/ftox.2023.1155645 (PMC10188990; doi:10.3389/ftox.2023.1155645)
Supplement: Supplementary file 1 [file Table1.DOCX]

**Supplemental Materials (SM)**

**SM1 Dose selection**

Preliminary experiments were performed to determine the concentration range for each compound.

By using the P.R.I.T.® ExpoCube®, NHBE cells (Human Bronchial/Tracheal Epithelial Cells, Lonza) were exposed to 4 different concentrations for 1 hour (acute exposure), starting with the highest achievable concentration according to the chemical-physical properties of the compound. Cell viability was then assessed using the WST-1 assay, a colorimetric assay to quantify cell viability. The concentration above 70% viability was used as the maximum concentration for the experiments with primary human bronchial epithelial cells (PBECs). Based on preliminary testing, 5 concentrations per compound were tested, only 2,5-hexanedione was tested in 4 concentrations due to limited volatility.


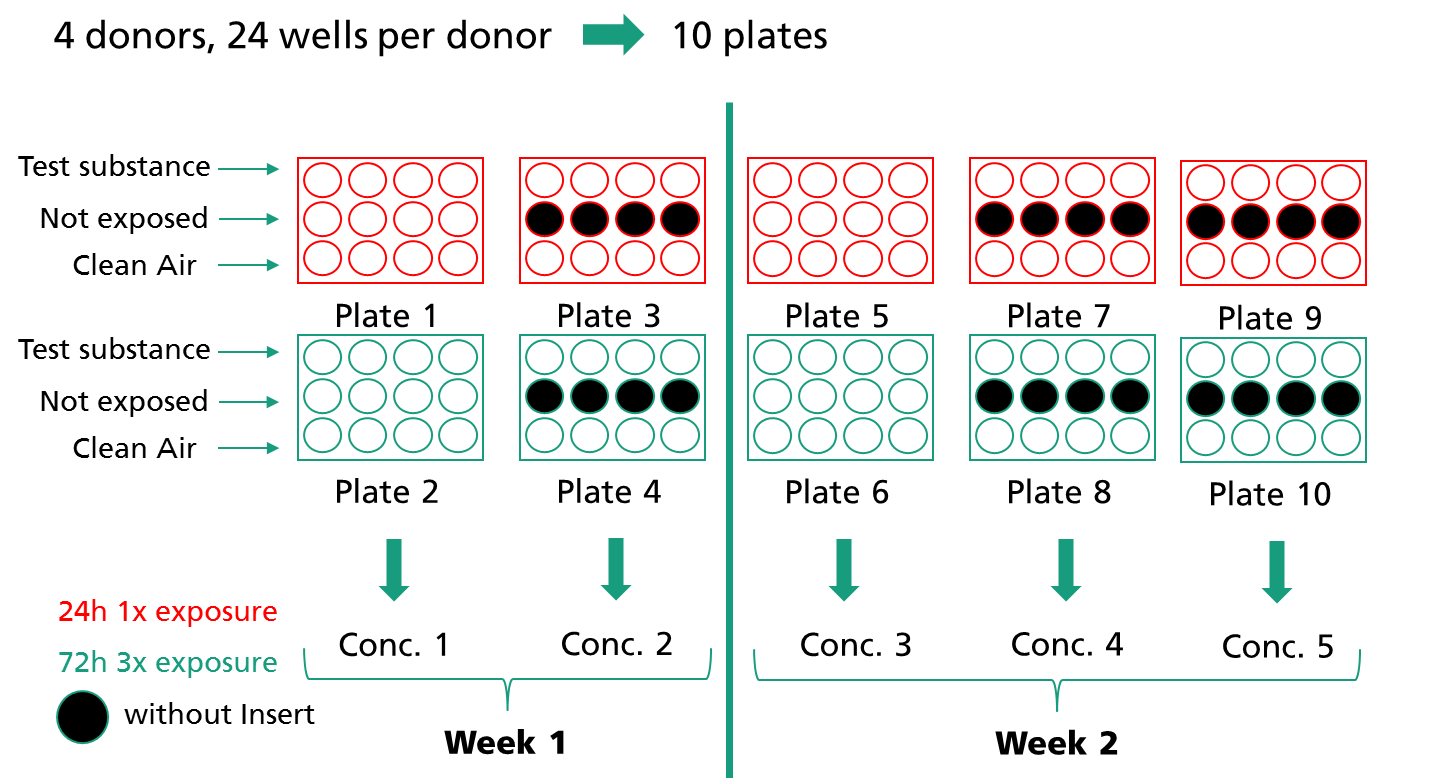


*Supplemental Figure 1:*  Experimental setup for week one and two of the ALI experiments. Primary human bronchial epithelial cells (PBECs) isolated from four different donors were cultivated in 12-well plates and exposed in three rows. The respective substance was exposed in the first row, the second row was not exposed and the third row was exposed to clean air.


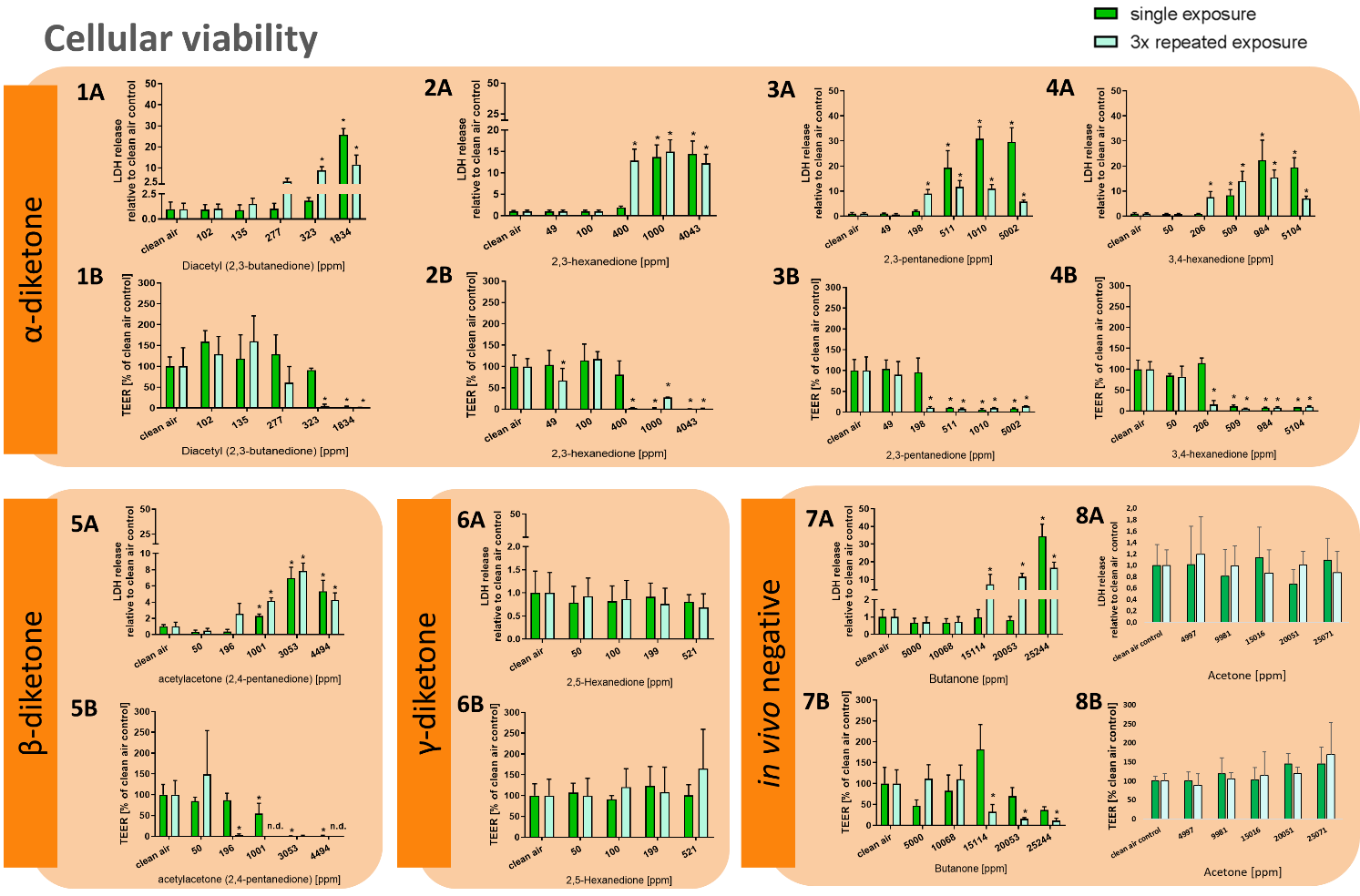


Supplemental Figure 2: The cellular viability was determined using LDH (A) and TEER (B) assays. Primary human bronchial epithelial cells (PBECs) isolated from four different donors were exposed for 1h once (single exposure) or repeatedly on three consecutive days (3x repeated exposure) to increasing concentrations [ppm] of the tested compounds. LDH release (A) and TEER values (B) are presented in % of clean air control and were measured 24h or 72h after the first exposure; *p<0.05 versus clean air control by one-way ANOVA followed by Tukey’s test.
